# Relationship of Temperament and Character in Remitted Depressed Patients with Suicidal Ideation and Suicide Attempts—Results from the CRESCEND Study

**DOI:** 10.1371/journal.pone.0105860

**Published:** 2014-10-03

**Authors:** Young Sup Woo, Tae-Youn Jun, Yang-Hwan Jeon, Hoo Rim Song, Tae-Suk Kim, Jung-Bum Kim, Min-Soo Lee, Jae-Min Kim, Sun-Jin Jo

**Affiliations:** 1 Department of Psychiatry, The Catholic University of Korea School of Medicine, Seoul, Korea; 2 Department of Psychiatry, Keimyung University School of Medicine, Daegu, Korea; 3 Department of Psychiatry, College of Medicine, Korea University, Seoul, Korea; 4 Department of Psychiatry, Chonnam National University Medical School, Gwangju, Korea; 5 Department of Preventive Medicine, The Catholic University of Korea School of Medicine, Seoul, Korea; Federal University of Rio de Janeiro, Brazil

## Abstract

The aim of this study was to evaluate the Temperament and Character Inventory (TCI) scores of a sample of Korean patients with remitted depression who had attempted suicide and reported suicidal ideation and to compare their scores with those of remitted depressed patients without suicidal ideation. Adult depression patients who had completed 12 weeks of follow-up (*N* = 138) were divided into three groups: patients with a history of suicide attempts (*N* = 23); patients with current suicidal ideation (*N* = 59); and patients without current suicidal ideation (*N* = 56). After controlling for covariates, no significant differences were found among the three groups on any measure of temperament or character except self-directedness and self-transcendence. The self-transcendence scores of the lifetime suicide-attempt group were significantly higher compared with those of the suicidal-ideation group; *post hoc* analysis revealed that self-directedness was significantly lower in the suicide-attempt group compared with the non-suicidal group. The results from the present study suggest that remitted depression patients with a history of suicide attempts do not differ from non-attempters in temperament, but do differ in certain character traits.

## Introduction

Suicide is a major public health problem worldwide, especially in patients with depression. The World Health Organization (WHO) estimates that 873,000 people commit suicide each year worldwide. This represents 1.4% of the total global burden of disease [1]. 60–70% of suicide victims have severe depression prior to committing suicide [2]. Moreover, as many as two-thirds of people with depression experience suicidal ideation, and 10–15% commit suicide [3]. The suicide rate in South Korea is increasing: the average number of daily deaths was 38.8 [4]. Korea also had the highest rate of suicide among the Organization for Economic Cooperation and Development countries in 2011 [5].

Although a variety of risk factors for suicidal behavior have been identified among persons with depression, including prior suicide attempts, hopelessness, co-morbid alcohol/substance abuse, further depressive episodes, and earlier age of onset of a major depressive disorder (MDD) [6]–[9], risk prediction is known to be imprecise. Indeed, false-positive and -negative findings are common [10], [11]. To predict and prevent suicidality, it is important to understand which personality traits that may be linked to suicidal behavior, and whether suicidality develops in the context of a time-limited psychiatric disorder or as part of a situation-dependent process [12]. Certain predisposing and protective factors for suicidal behavior lie in the structure of personality.

In several previous studies, personality, as represented in Cloninger's temperament and character model and measured by the Temperament and Character Inventory (TCI), has been investigated in suicidal patients. TCI is a widely used inventory that evaluates four major dimensions of temperament: harm avoidance (HA), novelty seeking (NS), reward dependence (RD), and persistence (PE), together with three major character dimensions: self-directedness (SD), cooperativeness (CO), and self-transcendence (ST) [13]. Temperament, as understood in this model, has been inferred largely from genetic studies of personality in humans and neurobiological studies with rodents [14]. The dimensions of temperament have been hypothesized to relate to brain neurotransmitter systems (where HA relates to serotonergic function, RD to noradrenergic function, and NS to dopaminergic function) [13].

Two temperament traits have been repeatedly associated with suicidal behavior: higher NS [15]–[18] and higher HA [19]–[22]. Other personality traits, including low RD, SD, and CO and high ST [20], [22]–[24], are also associated with suicidal behavior. However, previous studies have primarily compared psychiatric patients with a history of suicide attempts either to patients without a history of suicide attempts or to healthy controls. All of these studies evaluated TCI during the acute phase of the patients' illnesses; hence, TCI scores may have been affected by patients' mood. [19], [25], and depressed mood has been shown to be associated with higher HA scores and lower SD and CO scores [26]–[28].

To date, no study has specifically compared remitted depression patients who have attempted suicide to patients with and without suicidal ideation in the context of Cloninger's temperament and character model as measured by the TCI. The aim of the present study was to compare the personality traits, indexed by TCI scores, of Korean patients with remitted major depressive disorder across three groups: patients with a history of suicide attempts (suicide-attempt group); patients with current suicidal ideation but without a history of suicide attempts (suicidal-ideation group); and patients with neither current suicidal ideation nor a history of suicide attempts (non-suicidal group).

## Materials and Methods

The present study used data from the ongoing Clinical Research Center for Depression study (CRESCEND), whose design has been detailed elsewhere [29]. A summary of the study design is presented below.

### 1. Subjects

Between January 2006 and August 2008, CRESCEND enrolled 1,183 patients with depressive disorders (major depressive disorder, dysthymic disorder, and depressive disorder not otherwise specified) from 18 South Korean hospitals (16 university hospitals and two general hospitals). Enrolment occurred in a naturalistic clinical environment from both outpatient and inpatient settings irrespective of depression subtype or physical co-morbidities. The study was approved by all of the relevant university and/or hospital institutional review boards and was conducted according to the Declaration of Helsinki and good clinical practices. All participants reviewed the consent form, and written informed consent was obtained by research staff prior to their participation.

### 2. Inclusion and exclusion criteria

All patients with depressive disorders reviewed at the study hospitals were approached regarding participation. The clinicians assessing and diagnosing the patients applied the Diagnostic and Statistical Manual of Mental Disorders, Fourth Edition (DSM-IV) criteria [30]. CRESCEND used broad inclusion and minimal exclusion criteria in an effort to reflect the actual clinical situation in Korea. The inclusion criteria were (i) outpatients and inpatients >7 years of age and (ii) a DSM-IV diagnosis of depressive disorder (MDD with or without psychotic features, dysthymic disorder, or a depressive disorder not otherwise specified). The exclusion criteria were as follows: (i) a current or lifetime DSM-IV diagnosis of co-morbid schizophrenia, other psychotic disorder, bipolar disorder, organic psychosis, or dementia; (ii) a medical or neurologic illness of sufficient severity to interfere with the evaluations and interviews of the study; and (iii) pregnant or breastfeeding women. For analysis purposes, we selected only adult patients (≥20 years of age) who were in remission week 12, when this study was conducted, and excluded patients with other clinically diagnosed Axis I or II co-morbidities. Remission was determined by a Hamilton Depression Rating Scale (HAMD) [31] score of ≤7.

### 3. Assessments

The CRESCEND research protocol recommended assessments at baseline, 1, 2, 4, 8, 12, 24, and 52 weeks and annually thereafter. The analysis described herein was restricted to data obtained at baseline and week 12 because the TCI was only completed at week 12. Socio-demographic and clinical characteristics were evaluated at baseline. Baseline and week 12 scores were used in this analysis.

### 4. Determination of suicidality

We categorized patients into three groups: patients without current suicidal ideation and without a history of suicide attempts (non-suicidal group); patients with current suicidal ideation but without a history of suicide attempts (suicidal-ideation group); and patients with a history of suicide attempts with or without current suicidal ideation (suicide-attempt group). To determine suicidality, we investigated patients' suicide attempt histories, and we administered the Beck Scale for Suicide Ideation (SSI-B) [32] to measure current suicidal ideation. The participants and their families were asked whether patients had ever attempted suicide. A suicide attempt was defined as self-destructive behavior with at least some intent to die [33]. The SSI-B, a structured, self-administered scale, was used to identify patients with current suicidal ideation. A rating of “0” for item 4 indicates no active suicidal intent, and for item 5, indicates no avoidance of death if presented with a life-threatening situation. Thus, patients with suicidal ideation are rated as having either active or passive thoughts about killing themselves [34].

### 5. Statistical Analysis

Descriptive statistics are provided for socio-demographic data and psychometric tests. The chi-square test, Fisher's exact test, and analysis of variance (ANOVA) were conducted to compare the groups across categorical and continuous variables. The three groups' TCI scores were compared via analysis of covariance (ANCOVA), with sociodemographic variables that were significant at baseline and clinical variables significant at week 12 used as covariates. If two or more significant variables exhibited strong correlations (*p*<0.1), we excluded variables with low variance and/or significance. Finally, a *post hoc* analysis was performed to compare the three groups. Statistical significance was set at *p*<0.05 for all tests. All statistical analyses were conducted using the Statistical Analysis System software package (SAS, version 9.1; SAS Institute, Inc., Cary, NC, USA).

## Results

Of 1,183 total participants, 253 (21.4%) patients who had attempted suicide and 407 (34.4%) other patients completed follow-up to the week 12 visit. Information from the TCI was available for 325 patients (79.9%); 138 patients (42.5%) met the inclusion and exclusion criteria for the analysis described herein. The majority of the patients were female (*n* = 138; 85.5%), and 23 patients (16.7%) had a history of suicide attempts. Included patients were more likely to be employed and had lower baseline scores on the CGI-S, HAMD, BDI, and SSI-B and higher scores on the SOFAS and WHOQOL compared with the excluded patients (data not shown).

The baseline socio-demographic and clinical characteristics of the non-suicidal, suicidal-ideation, and suicide-attempt groups are summarized in Table 1. There were no significant differences in socioeconomic status, years of education, employment status, family history of depression or other psychiatric illnesses, current physical co-morbidities, or history of adverse life events among groups. There were significant differences among the three groups with respect to age (*df* = 2, *F* = 7.316, *p* = 0.001), age of onset (*df* = 2, *F* = 3.262, *p* = 0.046), marital status (*df* = 2, *χ* ^2^ = 10.736, *p* = 0.005), and religious status (*df* = 2, *χ* ^2^ = 7.278, *p* = 0.026). Among the baseline psychiatric symptom scales, the HAMA (*df* = 2, *F* = 5.721, *p* = 0.004), BDI (*df* = 2, *F* = 5.625, *p* = 0.005), WHOQOL-BREF (*df* = 2, *F* = 5.497, *p* = 0.005), and SSI-B (*df* = 2, *F* = 33.161, *p*<0.001) differed significantly across the three groups.

**Table 1:** Socio-demographic and clinical characteristics.

	Non suicidal group (*N* = 56)		Suicidal-ideation group (*N* = 59)		Suicide-attempt group (*N* = 23)		
	Mean (*N*)	*SD* (%)	Mean (*N*)	*SD* (%)	Mean (*N*)	*SD* (%)	*p*-value
Age (years)	50.7	15.6	49.1	16.5	36.2	14.8	0.001*
Female gender	45	80.4	53	89.8	20	87.0	0.379
Age at onset (years)	38.3	12.1	42.3	19.0	28.3	14.5	0.046*
Education (years)	10.6	4.7	9.9	4.5	11.3	4.0	0.423
Number of previous depressive episodes	6.2	21.9	1.9	2.1	1.5	1.5	0.488
Duration of illness	8.1	8.5	6.7	7.2	9.2	5.7	0.671
Currently married	42	75.0	43	72.9	9	39.1	0.005*
Living alone	52	92.9	51	86.4	19	82.6	0.328
No religious affiliation	13	23.2	15	25.4	12	52.2	0.026*
Annual household income, US$>19,000	31	55.4	31	52.5	12	52.2	0.944
Currently unemployed	11	19.6	8	13.6	4	17.4	0.684
Previous history of depression	20	35.7	23	39.0	13	56.5	0.219
Family history of depression	11	19.6	9	15.3	3	13.0	0.788
Family history of other psychiatric illness	6	10.7	8	13.6	1	4.3	0.583
Adverse life event	11	19.6	12	20.3	8	34.8	0.299
Current physical disorder	13	23.20	18	30.50	7	7	0.648
Baseline assessment scale scores							
CGI-S	4.4	1.1	4.3	1.1	4.6	1.0	0.558
HAMD	16.7	6.0	19.0	5.4	18.8	7.2	0.096
HAMA	13.7	5.8	17.3	7.0	18.3	7.8	0.004*
BDI	21.5	9.5	28.5	11.3	26.7	11.4	0.005*
SSI-B	2.8	3.2	9.9	6.6	15.0	8.3	0.000*
WHOQOL-BREF	69.0	8.8	65.3	9.3	61.7	8.5	0.005*
SOFAS	60.3	10.3	62.1	11.0	56.4	13.2	0.115
Week 12 assessment scale scores							
HAMD	3.8	2.3	3.8	2.0	4.4	2.4	0.487
HAMA	3.5	2.5	4.7	3.0	6.0	5.6	0.009*
CGI-S	1.7	0.9	1.8	0.8	2.1	1.2	0.174
BDI	7.0	6.5	9.2	8.7	8.7	8.4	0.315
SSI-B	1.3	2.6	2.8	5.1	5.3	5.9	0.002*
WHOQOL-BREF	70.6	10.7	68.4	12.1	69.1	12.5	0.591
SOFAS	78.3	10.0	78.1	7.3	76.3	7.8	0.614

CGI-S = Clinical Global Impression Scale; HAMD = Hamilton Depression Rating Scale; HAMA = Hamilton Anxiety Rating Scale; BDI = Beck Depression Inventory, SSI-B = Beck Scale for Suicidal Ideation; SOFAS = Social and Occupational Functioning Assessment Scale; WHOQOL-BREF = World Health Organization Quality of Life assessment instruments.

*p<0.05.

*Post hoc* analysis indicated that the suicide-attempt group was significantly younger compared with the suicidal-ideation (*p* = 0.004) and non-suicidal (*p* = 0.001) groups. Age of onset was higher in the suicidal-ideation group compared with the suicide-attempt group (*p* = 0.042). Patients in the suicide-attempt group were more likely to be unmarried/divorced/widowed (*p* = 0.005) and to have no religious affiliation (*p* = 0.026) compared with the other groups. Compared with the non-suicidal group, the baseline HAMA total score was higher in the suicidal-ideation (*p* = 0.015) and suicide-attempt (*p* = 0.019) groups. Baseline total BDI scores were higher in the suicidal-ideation group compared with the non-suicidal group (*p* = 0.004), and baseline WHOQOL scores were significantly lower in the suicide-attempt group compared with the non-suicidal group (*p* = 0.005).

At week 12, the total HAMD, BDI, CGI-S, WHOQOL-BREF, and SOFAS scores were not significantly different across groups. However, total HAMA scores were higher in the suicide-attempt group compared with the non-suicidal group (*p* = 0.009).

In the second phase of the analysis, ANCOVA was performed to compare group TCI scores at week 12, with age, gender, and week 12 HAMA total scores used as covariates. Age of onset, marital status and religion were excluded because they were correlated with age (data not shown), although they were significantly different across groups at baseline (Table 2). There were no significant group differences in scores for any TCI item. Based on the results of the *post-hoc* analysis, there were no significant differences between the three groups in temperament and character dimensions, with the exception of ST and SD (Table 2). ST scores in the suicidal-ideation group were significantly lower compared with the suicide-attempt group (*p* = 0.035), and the SD scores of the suicide-attempt group were significantly lower compared with the non-suicidal group (*p* = 0.033).

**Table 2:** Temperament and character inventory scores (ANCOVA with covariates of age, gender, and week 12 Hamilton Anxiety Rating Scale total score).

	Group 1 Non-suicidal (*N* = 56)		Group 2 Suicidal ideation (*N* = 59)		Group 3 Suicide attempts (*N* = 23)				Group 1 vs. 2	Group 1 vs. 3	Group 2 vs. 3
	mean	*SD*	mean	*SD*	mean	*SD*	*F*	*P*	*Post hoc* comparison		
Novelty seeking	22.0	4.6	22.8	5.2	23.5	5.1	0.049	0.953	0.805	0.958	0.796
Harm avoidance	13.0	7.4	14.2	5.8	14.0	7.0	0.709	0.494	0.557	0.259	0.43
Reward dependence	10.2	4.2	9.0	3.1	9.2	3.1	0.434	0.649	0.358	0.556	0.675
Persistence	3.6	2.1	3.5	1.7	4.0	1.8	1.976	0.143	0.911	0.156	0.067
Self-directedness	24.1	9.6	19.4	5.8	17.6	6.5	2.281	0.106	0.118	0.035*	0.388
Cooperativeness	16.0	9.3	13.6	6.9	13.1	5.7	0.237	0.789	0.496	0.576	0.892
Self-transcendence	19.0	5.9	17.9	6.3	20.1	5.2	2.451	0.09	0.817	0.189	0.033*

*p<0.05.

## Discussion

The aim of present study was to evaluate patients who were currently in remission from MDD, with or without suicidal ideation and prior suicide attempts, to identify a distinct personality profile for suicide risk based on the TCI. We compared TCI scores in remitted MDD patients with and without suicidal ideation and in patients who had attempted suicide. The suicide-attempt group had higher ST scores compared with the suicidal-ideation group and had lower SD scores compared with the non-suicidal group. Interestingly, there were no significant group differences in HA, NS, RD, CO, and PE scores among groups.

Several previous studies have reported that high HA, NS, and ST and low SD scores were associated with the risk for suicide [19], [24], [35]. Some researchers have suggested that the association between HA and suicidal behavior is questionable because the association may be dependent on underlying psychopathology and diagnostic status [24], [28], [36], [37]. Studies that have reported high NS and HA in suicidal patients have included patients with various diagnoses; furthermore, a study that only included patients with MDD did not exclude patients with Axis I or II co-morbidities [15], [16], [20], [24]. Moreover, the previous studies evaluated TCI in patients who were symptomatic for depression. Hirano et al. [27] reported that the level of depression was positively correlated with HA score and, further, that scores for HA changed significantly toward normal values in treatment responders, but were stable in treatment non-responders. In a recent meta-analysis, HA scores decreased with effective treatment in patients with MDD [14]. Therefore, high HA scores in the suicidal risk groups of previous studies may have been confounded by the presence of current depressive symptoms. Although the analysis controlled for severity of depression, there was a significant difference in such severity between groups [19]. Moreover, the inclusion criteria applied in the present study, which included only remitters, may have excluded a possible confounder in that high HA scores have previously been associated with increased risk of treatment-resistant depression [14]. With respect to NS, some discrepancies have been found in the direction of the association between NS and suicidality. Many studies have suggested a protective effect of NS [38], [39]; however, Brezo et al. [35] found only weak support for the importance of impulsivity and aggression in suicidal ideation, personality traits were mainly measured by the NS and CO dimensions of the TCI [13], [36].

In the present study, the suicide-attempt group had higher ST scores compared with the suicidal-ideation group and lower SD scores compared with the non-suicidal group. In a study with schizophrenic patients, high ST was associated with previous suicide attempts [40], and suicidality has been found to be associated with high ST in many other previous studies [15], [19], [23], [41]. This is in line with the results from the present study. SD subsumes personality features such as responsibility, resourcefulness, and self-acceptance [36]. Low SD has been linked to lack of conviction, which is important for problem solving, and to vulnerability to the environment [13]. Previous studies have reported a correlation between low SD and severity of psychiatric symptomatology [42]–[44]. Moreover, low SD is the principal TCI correlate of Axis II pathology [45]–[47]. Because we included remitted patients without Axis I or II comorbidity, our results suggest that low SD is related to suicidality and not to the severity of depressive symptoms and personality problems. This result is in accordance with previous studies that reported had lower SD among suicide attempters compared with non-attempters [15], [22], [44], [48]. Moreover, the relatively high ST and low SD scores of suicide attempters in the present study should be viewed in the context of previous studies' reports of an association between high ST and suicidality, as high ST is associated with low SD and is characterized by illogical, immature, and suspicious behavior [15], [41].

It is unclear why suicidal ideation was observed so frequently (42.8%, *n* = 59) in remitted depressive patients in the present study. However, the widely accepted relationship between impulsivity and suicidality [49], [50] may provide an explanation. In recent studies, Ekinci et al. [51], [52] investigated impulsivity, temperament, and character in euthymic patients with major depressive disorder. They reported that impulsivity scores were higher in remitted depressive patients compared with a healthy control group, and also that elevated impulsivity scores were associated with a history of suicide attempts. Accordingly, trait impulsivity may continue to influence suicidal ideation in remitted depressive patients. Moreover, there is evidence of a close relationship between trait anxiety and trait impulsivity [53], [54], which could also be an important risk factor for suicide ideation [55]. The higher HAMA score at week 12 in the suicidal-ideation and suicide-attempt groups, compared with the non-suicidal-ideation group, may reflect group differences in impulsivity.

The present study had a number of strengths. First, the study used data from the CRESCEND study, which is the largest-scale depression cohort study ever conducted in Asia and includes a wide range of data pertaining to the socio-demographic and clinical characteristics of depressed Korean patients. Second, we excluded depressed patients with psychiatric comorbidities to control for the confounding effects of personality disorders or other Axis I disorders, such as anxiety disorders, eating disorders, and substance misuse-related disorders. Third, we included only patients who were in remission from depressive episodes, and assessed TCI at week 12 to control for the possible effects of depressive symptom severity.

The present study also has several limitations. First, we included only Korean patients because cultural issues may lead to variation when assessing dimensions of temperament or character [26]. In a meta-analytic study comparing Cloninger's temperament dimensions across 20 countries [56], differences were particularly apparent between Asian and Western countries. The lowest mean score for RD was observed in the Japanese sample, and the highest mean score in PE was observed in the US sample. Moreover, the Asian sample had lower scores compared with other countries for NS, RD, and PE, and higher scores for HA. Inconsistency in the present results compared with previous studies reporting an association between suicidality and high HA and NS scores may be due to the low scores for HA and NS seen in the Korean sample. This is likely to reduce generalizability. Second, clinical diagnosis was a feature of the present study: this limits the reliability of the diagnosis of depression and other possible comorbid psychiatric disorders. Use of structured diagnostic interview schedules may increase the reliability and validity of diagnoses. Third, given the size of our samples, we could not stratify patients who had attempted suicide by the severity of their suicide attempts. Fourth, the patients included in our analysis had different baseline characteristics compared with excluded patients. Included patients were more likely to be female and employed, and they had lower baseline scores on the CGI-S, HAMD, BDI, and SSI-B and higher scores on the SOFAS and WHOQOL compared with excluded patients. In a previous study investigating TCI in remitted depressive patients [51], socio-demographic variables, including education, age, and gender, did not significantly contribute to TCI scores. However, the inclusion of patients with milder depressive symptoms and a higher degree of functioning, may limit the generalizability of the results of this study. Furthermore, detailed information pertaining to treatment was not included. Multiple, uncontrolled treatments can limit the interpretative validity of results. In a previous study, persistent suicidality was associated with treatment with selective serotonin reuptake inhibitors, a higher baseline SSI-B score and no HAMD- or HAMA-indexed remission episodes [57]. The present results should be interpreted in this context. Finally, certain TCI scores are likely to change over time [36]. We could not investigate changes in TCI scores in an attempt to limit this bias because TCI was only administered at week 12.

In conclusion, we did not observe differences among patients who attempted suicide and those with and without suicidal ideation in any of the dimensions of the TCI, with the exception of ST and SD. Longitudinal studies that include younger age groups are required to disentangle the personality profile of individuals with high suicidality from underlying psychopathology. The findings of the present study may further the delineation of this complex phenotype.
